# Gallstone Disease and the Risk of Cardiovascular Disease

**DOI:** 10.1038/s41598-019-42327-2

**Published:** 2019-04-09

**Authors:** Cameron J. Fairfield, Stephen J. Wigmore, Ewen M. Harrison

**Affiliations:** Department of Clinical Surgery, The University of Edinburgh, Edinburgh, EH16 4SA UK

## Abstract

Gallstone disease (GD) is one of the most common presentations to surgical units worldwide and shares several risk factors with cardiovascular disease (CVD). CVD remains the most common cause of death worldwide and results in considerable economic burden. Recent observational studies have demonstrated an association between GD and CVD with some studies demonstrating a stronger association with cholecystectomy. We present the findings of a meta-analysis assessing the relationship between GD and CVD. A total of fourteen cohort studies with over 1.2 million participants were included. The pooled hazard ratio (HR, 95% confidence interval [CI]) for association with GD from a random-effects model is 1.23 (95%CI: 1.16–1.30) for fatal and non-fatal CVD events. The association was present in females and males. Three studies report the relationship between cholecystectomy and CVD with a pooled HR of 1.41 (95%CI: 1.21–1.64) which compares to a HR of 1.30 (95%CI: 1.07–1.58) when cholecystectomy is excluded although confounding may influence this result. Our meta-analysis demonstrates a significant relationship between GD and CVD events which is present in both sexes. Further research is needed to assess the influence of cholecystectomy on this association.

## Introduction

Cardiovascular disease (CVD) remains the most common cause of death worldwide^1–3^. Despite significant advances in the prevention and treatment of CVD, the societal and economic burden is considerable and continues to rise^4^. CVD is preventable and it is cost-effective to promote risk factor modification in those at risk of CVD^5^. Early identification of at-risk populations may allow these changes to be implemented at a time when they are most effective.

Gallstone disease (GD) forms one of the most common presentations to surgical units worldwide^6–8^ and patients with gallstones have a higher prevalence of risk factors for cardiovascular disease including obesity and elements of the metabolic syndrome^9,10^. The presence of gallstones is associated with atherosclerosis^11^ and patients with gallstones may be at high risk of progression to symptomatic CVD.

Several observational studies have been published reporting a link between GD and CVD although some studies have found no difference^12–22^. While recent meta-analyses have demonstrated an association between GD and CVD, there is significant variation in the effect size of contributing studies. No meta-analysis to date has adequately accounted for confounding and the heterogeneity remains unexplained^21,23,24^. Further uncertainty is present over the role of cholecystectomy in modifying the risk of CVD with some authors concluding that cholecystectomy may directly cause CVD^24^. Analyses of cholecystectomy and CVD have not sufficiently accounted for the presence of confounding variables such as gallstone severity and inflammation. It is likely that these factors account for any apparent association between cholecystectomy and CVD. An additional cohort study examining the relationship between screen-detected GD and CVD has also been published with assessment of the impact of cholecystectomy on CVD^22^.

We therefore undertook a systematic review with meta-analysis of longitudinal studies assessing the impact of GD on CVD. We performed additional analyses assessing the impact of cholecystectomy on this association.

## Results

### Search Results

**Figure 1:**
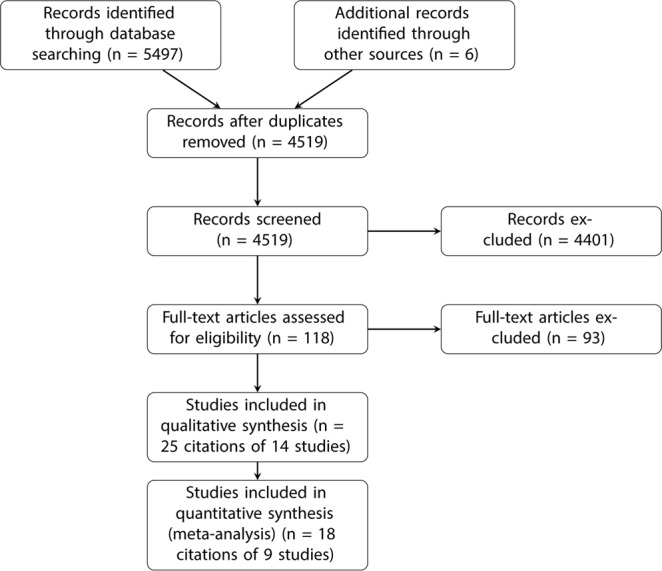
Flow chart of study selection process

The search identified 5497 references of which 984 were duplicates. Searching of bibliographies identified six other potentially relevant references. Following screening of titles and abstracts, 118 articles remained for full text review. After exclusion of 93 articles (not longitudinal studies, not including GD or not including CVD), a total of 25 citations referring to fourteen cohort studies were included in this systematic review (Fig. 1). One publication reported two cohort studies^14^ and one publication reported three separate cohort studies as well as a meta-analysis^21^. Two cohort studies included participants recruited from the same National Health Insurance Research Database (Taiwan) and recruited controls from overlapping inclusion periods^17,18^. To prevent bias in our meta-analysis we conducted all analyses twice: first using the larger of the two studies^18^ and second using the smaller study^17^. We present results from the first set of analyses and describe any differences from the second set of analyses.

### Study Characteristics

There were 14 prospective and retrospective cohort studies reported in eleven primary publications and reported in a further fourteen duplicate publications including conference abstracts. The characteristics of the studies are reported in Table 1

**Table 1:** Characteristics of Included Studies.

Author	Paul	Petitti	Bortnichak	Bortnichak	Grimaldi	Ruhl	Olaiya	Wei	Lv	Wirth	Zheng	Zheng	Zheng	Shabanzdeh
Year	1963	1979	1985	1985	1993	2011	2013	2014	2015	2015	2016	2016	2016	2017
Sex	Male	Female	Male	Female	Both	Both	Both	Both	Both	Both	Female	Female	Male	Both
Country	United States	United States	United States	United States	United States	United States	Taiwan	Taiwan	China	Germany	United States	United States	United States	Denmark
Design	Prospective cohort	Prospective cohort	Prospective cohort	Prospective cohort	Prospective cohort	Prospective cohort	Retrospective cohort	Retrospective cohort	Prospective cohort	Prospective cohort	Prospective cohort	Prospective cohort	Prospective cohort	Prospective cohort
Case ascertainment	Employees of WEC	WCCDS	FHS	FHS	GRIC	NHANES	NHIRD	NHIRD	CKB	EPIC-Germany	NHS	NHSII	HPFS	MONICA - Danish
GD diagnosis	Not stated	Not stated	Baseline US	Baseline US	Cholecystography	Baseline US	Inpatient insurance claims for GD	Inpatient HR	Self-reported GD at baseline	Self-reported GD at baseline	Self-reported GD at baseline	Self-reported GD at baseline	Self-reported GD at baseline	Baseline US
CVD diagnosis	Planned follow-up examination	Self-reported outcome verified in HR	HR	HR	Review of DC	Review of DC	National database linkage (ICD-9)	National database linkage (ICD-9)	Linkage to DC registry and insurance registry, active follow-up, verification of HR and DC by cardiologist	Postal questionnaire, HR or DC	HR and DC	HR and DC	HR and DC	National database linkage (ICD8 and ICD10)
CVD definition	CHD	CHD, cerebro-vascular disease, VTE	CHD, coronary insufficiency, sudden death, (not angina pectoris)	CHD, coronary insufficiency, sudden death, (not angina pectoris)	Death due to CVD	Death due to CVD	CHD, cerebrovascular disease, coronary insufficiency	Cerebrovascular disease	CHD	CHD, cerebrovascular disease	CHD	CHD	CHD	CHD, cerebro-vascular disease, VTE
No. Cases	Not stated	Not stated	180*	322*	222	2018	6981	135512	28345	4828	8796	5227	1449	504
No. Control	Not stated	Not stated	2028	2178	161	12210	27924	271024	459028	41658	103724	107692	42252	4992
Follow-up (years)	4.5	6.5	26	26	20	14.7	6	7–10	7.2	8.2	30	22	25	32
Con-founder adjust-ment	Not stated	Smoking, age, obesity, current OC use, previous OC use, hypertension, hypercholesterolaemia, alcohol consumption	Age, smoking, obesity, hypertension, hypercholesterolaemia, diabetes mellitus, LVH	Age, smoking, obesity, hypertension, hypercholesterolaemia, diabetes mellitus, LVH	Age, sex, diabetes mellitus, BMI, serum cholesterol	Age, sex, ethnicity, education, BMI, WHR, diabetes mellitus, HbA1c > 6.5%, serum cholesterol, smoking, alcohol consumption, caffeine consumption, CRP protein, SBP, DBP	Age, sex, PVD, hyperlipidaemia, diabetes mellitus, hypertension, COPD, alcoholism, CLD, HHA, AHA	Age, sex, hypertension, diabetes mellitus, IHD, AF, hyperlipidaemia	Age, sex, education, marital status, alcohol consumption, smoking, physical activity, red meat intake, fruit intake, vegetable intake, hypertension, diabetes mellitus, family history IHD, menopausal status, BMI, chronic hepatitis, cirrhosis, PUD	Age, sex, study centre, educational achievement, physical activity, smoking, alcohol intake, BMI, waist circumference, hypertension, hyperlipidaemia	Age, ethnicity, family history IHD, marital status, smoking, BMI, physical activity, diabetes mellitus, hypertension, hyperchole-sterolaemia, aspirin-use, alcohol intake, energy-adjusted cholesterol-intake, energy intake, menopausal status, HRT, OC use	Age, ethnicity, family history IHD, marital status, smoking, BMI, physical activity, diabetes mellitus, hypertension, hyperchole-sterolaemia, aspirin-use, alcohol intake, energy-adjusted cholesterol-intake, energy intake, menopausal status, HRT, OC use	Age, ethnicity, family history IHD, marital status, smoking, BMI, physical activity, diabetes mellitus, hypertension, hyperchole-sterolaemia, aspirin-use, alcohol intake, energy-adjusted cholesterol-intake, energy intake	Age, sex, cohort, BMI, SBP, DBP, non-HDL cholesterol, HDL, smoking, alcohol consumption, diet, physical activity, social group
Quality assessment (NOS)	Selection: 2 Comp-arability: 0 Outcome: 2	Selection: 2 Comp-arability: 1 Outcome: 2	Selection: 3 Comparability: 2 Outcome: 3	Selection: 3 Comp-arability: 2 Outcome: 3	Selection: 2 Comp-arability: 1 Outcome: 3	Selection: 3 Comp-arability: 2 Outcome: 3	Selection: 4 Comparability: 2 Outcome: 1	Selection: 4 Comparability: 1 Outcome: 1	Selection: 4 Comparability: 1 Outcome: 2	Selection: 2 Comparability: 2 Outcome: 1	Selection: 3 Comparability: 2 Outcome: 2	Selection: 3 Comparability: 2 Outcome: 2	Selection: 3 Comp-arability: 2 Outcome: 2	Selection: 4 Comp-arability: 2 Outcome: 3

GD: gallstone disease; CVD: cardiovascular disease; NOS: Newcastle-Ottawa Scale; US: ultrasound; WEC: Hawthorne Works of the Western Electrical Company (Chicago); WCCDS: Walnut Creek Contraceptive Drug Study; FHS: Framingham Heart Study; GRIC: Gila River Indian Community; NHANES: National Health and Nutrition Examination Survey; NHIRD: National Health Insurance Research Database; CKD: China Kadoorie Biobank; EPIC-Germany: European Prospective Investigation into Cancer and Nutrition, German cohorts; NHS: Nurses’ Health Study; NHSII: Nurses’ Health Study II; HPFS: Health Professionals Follow-up Study; MONICA: Multinational mONItoring of trends and determinants in CArdiovascular disease, Danish cohorts only), HR: hospital records; DC: death certificates; ICD-9-CM: International Classification of Diseases, 9th Revision, Clinical Modification; IHD: ischaemic heart disease; VTE: venous thromboembolism; MI: myocardial infarction; PVD: peripheral vascular disease; OC: oral contraceptive; LVH: left ventricular hypertrophy; BMI: body mass index; WHR: waist-to-hip ratio; CRP: C-reactive protein; SBP: systolic blood pressure; DBP: diastolic blood pressure; COPD: chronic obstructive pulmonary disease; CLD: chronic liver disease; HHA: hereditary haemolytic anaemia; AHA: acquired haemolytic anaemia; AF: atrial fibrillation; PUD: peptic ulcer disease; HRT: hormone-replacement therapy without cholecystectomy excluded from analysis, number of cases remaining in analysis not stated.

. Two studies reported CVD mortality, seven studies reported coronary heart disease (CHD), one study reported cerebrovascular disease and four studies reported multiple CVD outcomes. Four studies relied on baseline ultrasound for diagnosis of GD, one study used cholecystography, two studies used International Classification of Disease (ICD) database records of GD verified in hospital records, five studies used self-reported GD at baseline with four of these verifying reports through hospital records and two studies do not report on ascertainment of exposure to GD and define exposure as “gallbladder disease” rather than GD. Both of the studies which consider “gallbladder disease” do not provide adequate numeric data to be incorporated into a meta-analysis and are therefore considered only in the qualitative analysis. It was felt that “gallbladder disease” was most likely to represent gallstones in these studies given the relative rarity of other gallbladder pathologies although caution is needed when considering the findings of these two studies. All of the remaining studies define exposure as “gallstone disease”. Three studies report separate outcomes for participants with GD who do not undergo cholecystectomy compared to controls and participants who undergo cholecystectomy compared to controls. Five studies report the rate of cholecystectomy which varied from approximately 31% to 73%. Four studies included only females and three studies included only males. Length of follow-up varied from 4.5 to 32 years with a median of 20 years.

Nine studies report adjusted HR of which eight are included in the main analysis (one includes duplication of controls from the same database). Two studies reported in the same publication report odds ratio (OR) and are considered in the qualitative analysis. Two studies report relative risk (RR) of which one does not report a sample size and the studies were therefore considered qualitatively. One study does not report numeric data. Overall this review includes over 1,269,137 participants (number of participants not provided in two studies). The quantitative meta-analysis included 1,229,171 participants (186,589 cases and 1,042,582 controls).

### Gallstone Disease and the risk of Cardiovascular Disease

Ten of the fourteen studies reported a statistically significant increase in CVD due to GD. Three studies report non-statistically significant findings of which two demonstrate a trend towards an increase in CVD with GD and one reports a trend towards reduced CHD with GD in a female-only cohort. The study reporting no numeric data examined CHD and states: “There was no significant relationship with a history of gallbladder disease”.

#### All Cardiovascular Disease

**Figure 2:**
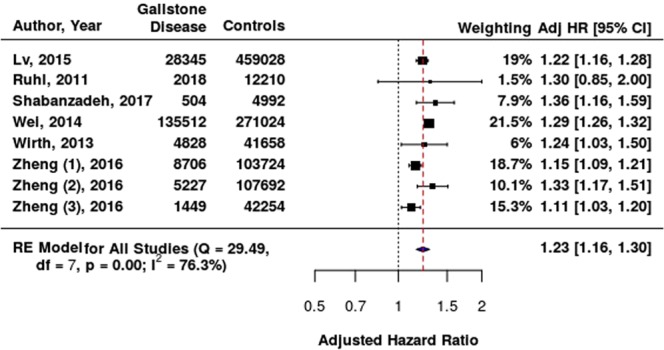
Forest plot demonstrating relationship between GD and CVD. Squares and horizontal lines represent individual studies with 95% confidence intervals. Area of squares is proportional to study weighting in random effects meta-analysis. Diamond (blue) represents pooled HR from meta-analysis with 95% CI. Reference line (black) represents hazard ratio of 1.00 (no association between GD and CVD). Summary line (red) represents summary estimate and demonstrates increased risk of CVD with GD (23%).

After controlling for confounding variables participants with GD had a 23% increased risk of CVD compared to controls (95% CI = 1.16–1.30, Fig. 2). We identified considerable heterogeneity (I^2^ = 76.3%, p = 0.0001).

#### Coronary Heart Disease

Seven studies reported CHD as a separate outcome including 843,312 participants (56,040 cases and 787,272 controls). Participants with GD had a 24% increased risk of CHD compared to controls (95% CI = 1.16–1.32). We identified considerable heterogeneity when CHD was considered (I^2^ = 70.7%, p = 0.0023).

#### Cerebrovascular Disease

Four studies reported cerebrovascular disease as a separate outcome although two studies used controls from the same database. Three studies were pooled by meta-analysis including 458,518 participants (140,844 cases and 317,674 controls). Participants with GD had a 29% increased risk of cerebrovascular disease compared to controls (95% CI = 1.26–1.32). We did not identify heterogeneity when cerebrovascular disease was considered (I^2^ = 0.00%, p = 0.76).

### Subgroup Analyses and Sensitivity Analyses

**Figure 3:**
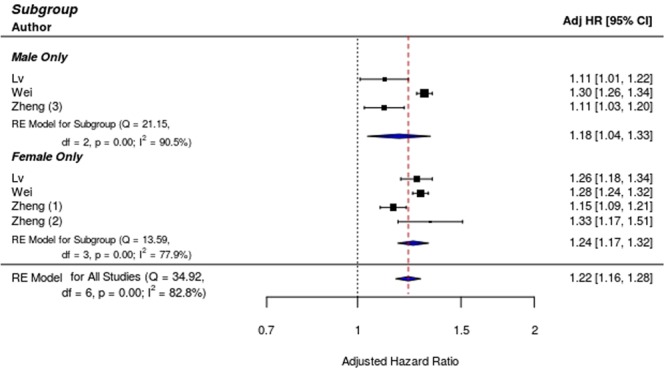
Forest plot demonstrating relationship between GD and CVD stratified by sex. Blue diamonds represent pooled summary estimate with 95% CI for each subgroup and overall pooled estimate for both groups combined.

We performed subgroup analyses for male-only participants, female-only participants, non-diabetic participants, diabetic participants, screen-detected GD and symptomatic GD (Table 2). We identified a statistically significant association between GD and CVD in all subgroups. One study which reported RR reported a summary estimate <1.00 but was not statistically significant and included only 322 cases compared to 103,562 cases included in our meta-analysis. We therefore demonstrate that the relationship between GD and CVD is present in both men and women (Fig. 3). The difference between diabetic and non-diabetic participants is not statistically significant although a trend towards a stronger effect suggests that GD may have a greater relative importance as a predictor of CVD in non-diabetic participants. CVD was significantly more common in both screen-detected and symptomatic GD. Heterogeneity remained moderate to substantial in all subgroup analyses other than cerebrovascular disease, screen-detected GD and cholecystectomy-only.

**Table 2:** Subgroup Analyses for relationship between GD and CVD.

Subgroup	HR (95% CI)	P (Cochran Q test)	I^2^	P (Wald-type test)	Studies
Sex					
Female	1.24 (1.17–1.32)	0.000	77.9%	0.428	5*
Male	1.18 (1.04–1.33)	0.000	90.5%		4*
Diabetes					
Diabetic	1.13 (1.06–1.20)	NA	NA	0.075	1
Non-diabetic	1.23 (1.15–1.32)	0.000	91.3%		6**
Detection of GD					
Screen-detected	1.35 (1.17–1.57)	0.847	0.00%	0.183	2
Symptomatic	1.21 (1.15–1.29)	0.000	82.4%		6*

Studies which included duplicated participants report subgroup results for male and female participants and symptomatic GD, we therefore conducted two separate analyses which produced similar results. studies reported in one publication are pooled by meta-analysis and both studies which included duplicated participants report subgroup analyses for non-diabetic participants and we therefore conducted four separate analyses (with and without inclusion of the pooled result and with and without each study using duplicated participants) which produced similar results.

Two studies from the same database report similar increases in CVD with GD for normotenstive and hypertensive participants. One study reports similar increases in CVD with GD for hyperlipidemic and non-hyperlipidemic participants. Three studies from the same publication were pooled in a meta-analysis for non-obese participants and for non-smokers revealing similar results in both analyses to the overall cohorts. Two studies from the same database report HRs for comorbidity-free participants as well as age-stratified subgroups for which results are similar to the overall cohorts except in the study assessing effect of GD on cerebrovascular where younger and comorbidity-free participants appear to have relatively greater burden of CVD risk. One study stratified participants according to GD duration at baseline and found no difference between groups.

We performed sensitivity analyses by iteratively excluding one study from the analysis to detect its impact on heterogeneity. Heterogeneity remained substantial for all sensitivity analyses with one study removed. Heterogeneity was noted to fall (I^2^ = 49%) when both Taiwanese studies using data from the same database were excluded.

We performed a meta-regression which did not detect any significant effect of follow-up, year of publication, quality assessment score, degree of adjustment for confounding variables or variance (p > 0.4 in all cases). In addition, none of the explanatory variables accounted for heterogeneity (pseudo-R^2^ = 0.00%, I^2^ = 70.4%).

### Effect of Cholecystectomy on risk of Cardiovascular Disease

Three studies report separate data for cholecystectomy compared to controls and for GD without cholecystectomy compared to controls. One study reports a separate analysis of cholecystectomy versus GD without cholecystectomy. Pooled adjusted HR for cholecystectomy compared to controls was 1.41 (95%CI: 1.21–1.64) with insignificant heterogeneity (p = 0.250, I^2^ = 27.8%). Pooled adjusted HR for GD without cholecystectomy compared to controls was 1.30 (95%CI:1.07–1.58) with moderate heterogeneity (p = 0.161, I^2^ = 45.3%). The study compared cholecystectomy to GD without cholecystectomy directly found no statistically significant difference (HR 1.24, 95%CI: 0.85–1.81). One study also reported mild GD (cholelithiasis or choledocholithiasis excluding definition of severe GD) and severe GD (acute calculous cholecystitis, calculous pancreatitis, cholangitis, surgical procedure for GD or endoscopic procedure for GD) and reported a stronger association in mild gallstone disease (HR 1.34, 95%CI: 1.24–1.46 for non-severe GD versus HR 1.20, 95%CI: 1.02–1.40 for severe GD).

One report including three cohort studies limited exposure to cholecystectomy only and stated: “CHD risk was similar to when our definition of gallstone disease included both history of gallstones and cholecystectomy” although does not provide numeric data for this subgroup^21^. One report including two cohort studies states: “Cholecystectomy status proved to be superior to gallstone disease diagnosis in discriminating between those individuals who went on to develop coronary disease and those who did not”. This report did not provide data for GD with and without cholecystectomy^14^.

Overall four studies favour a stronger relationship between GD and CVD when cholecystectomy alone is considered and six studies favour a weaker relationship or no difference. No studies report a statistically significant difference between the two groups. Only one study^16^ adjusted for inflammatory markers (C-reactive protein) and found a non-significant trend towards lower risk of CVD in patients undergoing cholecystectomy compared to those with gallstones who did not undergo cholecystectomy.

### Publication Bias

**Figure 4:**
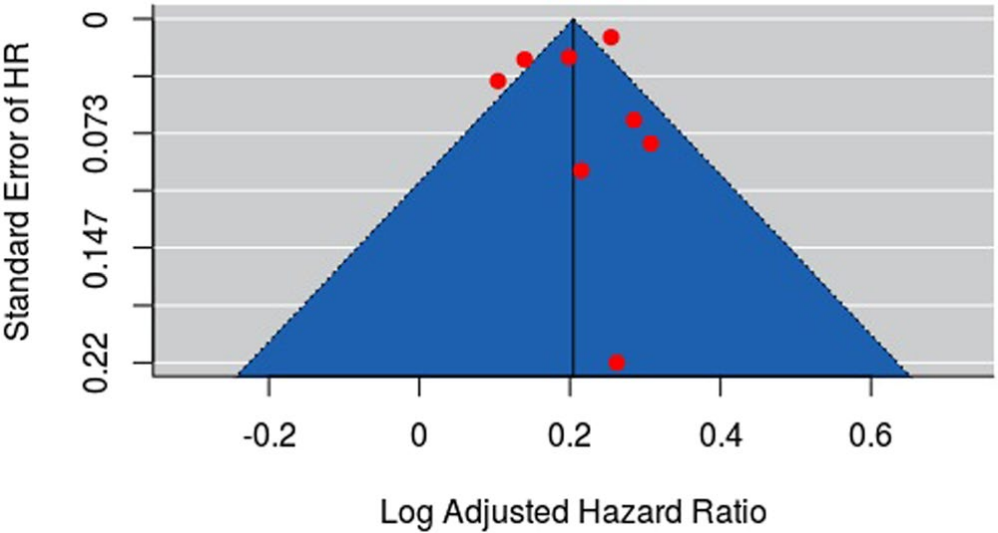
Funnel plot with pseudo-95% confidence limits. Circles represent identified studies. Log-transformed adjusted HR plotted against inverse standard error of log-transformed HR.

We did not detect publication bias through visual inspection of the funnel plot or by Egger’s regression test (p = 0.51, Fig. 4).

## Discussion

This systematic review with meta-analysis includes over 1.2 million participants and demonstrates a significant association between GD and CVD with an increase of 23% between cases and controls following adjustment for confounding variables. The association with GD is present for CHD, cerebrovascular disease and PVD as well as for men and women. There is trend towards a relatively stronger association in young and otherwise healthy individuals although no subgroup differences were statistically significant. The review highlights a scarcity of high quality evidence for the association between cholecystectomy and CVD.

Three separate meta-analyses investigating the relationship between GD and CVD have been published and report similar findings to our review with a statistically significant increase in CVD with GD which is present in several subgroups^21,23,24^.

Possible mechanisms linking GD and CVD include dyslipidemia, chronic inflammation and abnormalities in the ABCG8 cholesterol transporter gene^25–27^. Patients with the D19H and T400k polymorphisms of the ABCG8 gene have been demonstrated to have elevated risk of both CVD and GD suggesting a shared aetiology through aberrant cholesterol metabolism^28,29^. The presence of gallstones is known to be associated with inflammation of the gallbladder mucosa^7^. The presence of inflammation is already known to promote formation of atherosclerotic plaques even at distant sites and it is possible that low-grade inflammation associated with gallstones triggers a similar response^30,31^. GD is also associated with obesity, hyperlipidemia, diabetes mellitus and hypertension^10,32^ which is responsible in part for an increase in CVD. Adjustment of the reported HR for these confounding variables results in a reduction of CVD risk although, in all but two studies the association remains statistically significant. Other aetiological mechanisms such as altered gut microbiota and reduced levels of serum insulin-like growth factor are implicated in gallstone development and development of atherosclerotic plaques that have not been accounted for in any existing studies^33–36^.

In another meta-analysis, Fan *et al*. conclude that cholecystectomy is likely to be responsible for increased risk of CVD compared to GD without cholecystectomy. They cite evidence of altered serum lipid levels post-cholecystectomy in animal models, protective effects of biliary hormones on lipid and glucose metabolism and post-cholecystectomy alteration in gut microbiota. We do not feel that the evidence is sufficient to support this hypothesis. Only one cohort study makes direct comparison between cholecystectomy and GD without cholecystectomy and finds no significant difference. Changing the definition of exposure from GD to cholecystectomy is reported to have no significant impact in three studies although numeric data is not provided. Two studies report a trend towards higher rates of CVD following cholecystectomy and one publication reporting two studies only published data for cholecystectomy as this was found to be a stronger predictor of CVD than GD as a whole. Three studies found a trend towards a weaker association between GD and CVD when cholecystectomy only was considered. Of the studies which find cholecystectomy to be a weaker predictor of CVD than GD as a whole, one of these studies^16^ adjusts for C-reactive protein suggesting that inflammation may be responsible for any apparent increase in CVD in the cholecystectomy group. None of the studies which demonstrate a trend towards higher rates of CVD in the cholecystectomy group, compared to those with gallstones who do not require cholecystectomy, adjust for inflammatory markers. Symptoms arising from inflammation of the gallbladder are a key determinant of the need for cholecystectomy, meaning that patients who require cholecystectomy may have suffered from more extensive systemic inflammation. For these reasons we do not feel that cholecystectomy contributes directly to increased risk of CVD and is, instead, more likely to represent those patients who have a greater burden from other CVD risk factors.

Our meta-analysis identified substantial heterogeneity for which we did not identify the likely cause. It is possible that additional confounding variables at participant- or study-level have not been recorded or reported. A total of five studies reported having additional data available, which we were unable to obtain, relating to differences between rate of CVD following cholecystectomy compared to GD without cholecystectomy. This suggests that the review may be subject to selective reporting bias with only those articles finding a difference between the two comparisons publishing the results. Our review identified a persistent direction of effect in all but one study and included data from three studies not previously considered in any other review on the same topic. The findings are consistent in several populations and subgroups but do not demonstrate a causal link between GD or cholecystectomy and CVD.

Our review has confirmed the significant association between GD and CVD and highlights the importance of recognizing GD as a risk factor for CVD. We have demonstrated that risk of CVD is elevated in GD for both males and females. We have demonstrated that cholecystectomy may not increase the risk of CVD as previously thought. Our review identifies the need for further research into the impact of chronic inflammation on the risk of CVD in patients with GD and in particular whether this accounts for any apparent increase in CVD with cholecystectomy.

## Methods

A systematic review with meta-analysis was undertaken in accordance with the Meta-analysis of Observation Studies in Epidemiology (MOOSE) statement^37^ and the PRISMA statement^38^.

### Search Strategy

In October 2017 we ran searches in MEDLINE, EMBASE, Science Citation Index and Social Sciences Citation Index Expanded and Literatura Latino-americana e do Caribe em Ciências da Saúde (LILACS) from earliest date possible. The search strategies are shown in Supplementary Table S1. We combined terms into three categories (GD; CVD; and observational studies) using the OR operator before combining the three categories using the AND operator.

### Study Selection

We included studies meeting the following criteria:

- Longitudinal cohort study
- Assessing the relationship between GD and CVD

We included cholecystectomy as well as GD without cholecystectomy, we also included screen-detected as well as symptomatic GD. Studies which reported “gallbladder disease” and were thought to represent gallstones rather than rarer gallbladder pathology were considered only in the qualitative analysis. We included fatal and non-fatal CVD events and considered CHD, cerebrovascular disease and peripheral vascular disease (PVD). Each subgroup of CVD was considered separately in subanalyses where possible as well as combined into one single composite outcome of CVD. No relevant non-English language articles were identified. When identified studies were reported in multiple publications we extracted unique data from each publication.

### Data Extraction

We extracted the following data from each study: authors, publication year, study population, baseline characteristics, sample size, study design, country, ascertainment of exposure, detection of CVD events, definition of CVD, follow-up duration, confounding variable adjustment, rate of cholecystectomy and adjusted effect measures (hazard ratio [HR], relative risk [RR] or odds ratio [OR]) with 95% confidence interval. We assessed each study according to the Newcastle-Ottawa Quality Assessment Scale (NOS)^39^. Prior to data extraction we defined an appropriate follow-up period of at least 10 years to allow for CVD events to occur and a follow-up rate of >90%. We contacted authors to request additional data when required.

### Data Analysis

We assimilated data using DerSimonian-Laird random effects meta-analysis^40^. We used fully adjusted (multivariable) effect measures from each study when available. We did not transform each unique effect measures into one single effect measure and as such studies reporting HR, RR or OR are considered in three distinct groups. All effect measures were log-transformed for purposes of data analysis prior to back-transformation for presentation of results.

Subgroup analyses were performed for the following groups:

- Male-only participants
- Female-only participants
- Diabetic participants
- Non-diabetic participants
- Screen-detected GD
- Symptomatic GD
- Gallstones (not including cholecystectomy) versus controls
- Cholecystectomy only versus controls

Discrete subgroups were compared using a Wald-type test with p value significance set at <0.01. We assessed influences of moderator variables by meta-regression using a mixed effects model and the Knapp and Hartung adjustment^41^. For the mixed effects model and meta-regression we included length of follow-up, year of publication, degree of adjustment for confounding variables, variance and NOS score.

We assessed for presence of heterogeneity using Cochran’s Q test with p value significance set at <0.1 and quantified heterogeneity with the I^2^ statistic^42,43^. We assessed for the presence of publication bias by visual inspection of funnel plots and Egger’s regression test with p value significance set at <0.1^44^.

All analyses were undertaken in R version 3.3.1 using the “metafor” package^45,46^. All data is available in original manuscripts.

## Supplementary information

Supplementary Information
