# Supplementary material for: Gallstone Disease and the Risk of Cardiovascular Disease
Source: Sci Rep. 2019 Apr 9;9:5830. doi: 10.1038/s41598-019-42327-2 (PMC6456597; doi:10.1038/s41598-019-42327-2)
Supplement: Supplementary file 1 — Supplementary Information [file 41598_2019_42327_MOESM1_ESM.pdf]

# Supplementary Information

## Gallstone Disease and the Risk of Cardiovascular Disease

Systematic Review and Meta-analysis of Observational Studies

Cameron J Fairfield[1]*, Stephen J Wigmore[1], and Ewen M Harrison[1]

[1]Department of Clinical Surgery, The University of Edinburgh, Edinburgh, EH16 4SA, UK

*Corresponding Author Email: cameron.fairfield@ed.ac.uk

Supplementary Table S1. Search Strategies

| Database | Time Span | Search Strategy |
|---|---|---|
| MEDLINE (OVID SP) | 1946 to October 2017 | #1 exp Gallstones/<br>#2 exp Cholecystectomy/<br>#3 exp Cholecystectomy, Laparoscopic/<br>#4 (gallstone* or gallbladder-calcul* or gallbladder-stone* or gall-bladder-stone* or gall-bladder-calcul or cholelith* or choledocholith* or cholesterol-stone*).mp. [mp=title, abstract, original title, name of substance word, subject heading word, keyword heading word, protocol supplementary concept word, rare disease supplementary concept word, unique identifier, synonyms]<br>#5 1 or 2 or 3 or 4<br>#6 exp Myocardial Ischemia/<br>#7 exp Myocardial Infarction/<br>#8 exp Coronary Disease/<br>#9 exp Stroke/<br>#10 exp Diabetes Mellitus, Type 2/<br>#11 exp Diabetes Mellitus/<br>#12 exp Metabolic Syndrome/<br>#13 exp Insulin Resistance/<br>#14 exp Peripheral Vascular Diseases/<br>#15 (myocardial-ischem* or myocardial-ischaem* or ischaemic-heart* or ischemic-heart* or myocardial-infarct* or angin* or acute-coronary* or ACS* or cardiac-infarct* or coronary-heart* or coronary-disease* or stroke* or cerebrovascular-disease* or cerebral-ischaem* or cerebral-ischem* or blood-gluc* or blood-sug* or serum-gluc* or serum-sug* or metabolic-syndro* or insulin-resist* or peripheral-vasc* or arterial-ulc* or peripheral-arter* or PAD* or PVD*).mp. [mp=title, abstract, original title, name of substance word, subject heading word, keyword heading word, protocol |

| | | |
|---|---|---|
| | | supplementary concept word, rare disease supplementary concept word, unique identifier, synonyms]<br>#16 Cohort Studies/<br>#17 Case-Control Studies /<br>#18 Cross-Sectional Studies/<br>#19 Longitudinal Studies/<br>#20 Retrospective Studies/<br>#21 Prospective Studies/<br>#22 Follow-up Studies/<br>#23 Incidence/<br>#24 Meta-analysis/<br>#25 ((retrospective* adj3 (cohort* or stud* or cross* or case* or longitudin* or follow* or review* or meta-analys*)) or (prospective* adj3 (cohort* or stud* or cross* or case* or longitudin* or follow* or review* or meta-analys*)) or cohort* or case-control* or cross-section* or longitudinal* or follow-up-stud* or review* or meta-analys* or incidence*).mp. [mp=title, abstract, original title, name of substance word, subject heading word, keyword heading word, protocol supplementary concept word, rare disease supplementary concept word, unique identifier, synonyms] |
| EMBASE and EMBASE CLASSIC (OVID SP) | 1947 to October 2017 | #1 exp Gallstones/<br>#2 exp Cholecystectomy/<br>#3 exp Cholecystectomy, Laparoscopic/<br>#4 (gallstone* or gallbladder-calcul* or gallbladder-stone* or gall-bladder-stone* or gall-bladder-calcul or cholelith* or choledocholith* or cholesterol-stone*).mp. [mp=title, abstract, subject headings, heading word, drug trade name, original title, device manufacturer, drug manufacturer, device trade name, keyword]<br>#5 1 or 2 or 3 or 4<br>#6 exp Myocardial Ischemia/<br>#7 exp Myocardial Infarction/ |

| | | |
|---|---|---|
| | | #8 exp Coronary Disease/<br>#9 exp Stroke/<br>#10 exp Diabetes Mellitus, Type 2/<br>#11 exp Diabetes Mellitus/<br>#12 exp Metabolic Syndrome/<br>#13 exp Insulin Resistance/<br>#14 exp Peripheral Vascular Diseases/<br>#15 (myocardial-ischem* or myocardial-ischaem* or ischaemic-heart* or ischemic-heart* or myocardial-infarct* or angin* or acute-coronary* or ACS* or cardiac-infarct* or coronary-heart* or coronary-disease* or stroke* or cerebrovascular-disease* or cerebral-ischaem* or cerebral-ischem* or blood-gluc* or blood-sug* or serum-gluc* or serum-sug* or metabolic-syndro* or insulin-resist* or peripheral-vasc* or arterial-ulc* or peripheral-arter* or PAD* or PVD*).mp. [mp=title, abstract, subject headings, heading word, drug trade name, original title, device manufacturer, drug manufacturer, device trade name, keyword]<br>#16 Cohort Studies/<br>#17 Case-Control Studies /<br>#18 Cross-Sectional Studies/<br>#19 Longitudinal Studies/<br>#20 Retrospective Studies/<br>#21 Prospective Studies/<br>#22 Follow-up Studies/<br>#23 Incidence/<br>#24 Meta-analysis/<br>#25 ((retrospective* adj3 (cohort* or stud* or cross* or case* or longitudin* or follow* or review* or meta-analys*)) or (prospective* adj3 (cohort* or stud* or cross* or case* or longitudin* or follow* or review* or meta-analys*)) or cohort* or case-control* or cross-section* or longitudinal* or follow-up-stud* or review* or meta-analys* or incidence*).mp. [mp=title, abstract, subject |

| | | |
|---|---|---|
| | | headings, heading word, drug trade name, original title, device manufacturer, drug manufacturer, device trade name, keyword] |
| Science Citation Index EXPANDED and Social Sciences Index | 1900 to October 2017 | #4 TS=#3 AND #2 AND #1<br>#3 TS=((retrospective* adj3 (cohort* or stud* or cross* or case* or longitudin* or follow* or review* or meta-analys*)) or (prospective* adj3 (cohort* or stud* or cross* or case* or longitudin* or follow* or review* or meta-analys*)) or cohort* or case-control* or cross-section* or longitudinal* or follow-up-stud* or review* or meta-analys* or incidence*)<br>#2 TS=(myocardial-ischem* or myocardial-ischaem* or ischaemic-heart* or ischemic-heart* or myocardial-infarct* or angin* or acute-coronary* or ACS* or cardiac-infarct* or coronary-heart* or coronary-disease* or stroke* or cerebrovascular-disease* or cerebral-ischaem* or cerebral-ischem* or blood-gluc* or blood-sug* or serum-gluc* or serum-sug* or metabolic-syndro* or insulin-resist* or peripheral-vasc* or arterial-ulc* or peripheral-arter* or PAD* or PVD*)<br>#1 TS=(gallstone* or gallbladder-calcul* or gallbladder-stone* or gall-bladder-stone* or gall-bladder-calcul or cholelith* or choledocholith* or cholesterol-stone*) |
| Literatura Latino Americana e do Caribe em Ciências da Saúde (LILACS) | Bireme; 1982 to October 2017 | (gallstone$ or gallbladder-calcul$ or gallbladder-stone$ or gall-bladder-stone$ or gall-bladder-calcul or cholelith$ or choledocholith$ or cholesterol-stone$) [words] and (myocardial-ischem$ or myocardial-ischaem$ or ischaemic-heart$ or ischemic-heart$ or myocardial-infarct$ or angin$ or acute-coronary$ or ACS$ or cardiac-infarct$ or coronary-heart$ or coronary-disease$ or stroke$ or cerebrovascular-disease$ or cerebral-ischaem$ or cerebral-ischem$ or blood-gluc$ or blood-sug$ or serum-gluc$ or serum-sug$ or |

| | | |
|---|---|---|
| | | metabolic-syndro$ or insulin-resist$ or peripheral-vasc$ or arterial-ulc$ or peripheral-arter$ or PAD$ or PVD$) [words] and (retrospective$ or prospective$ or cohort$ or case-control$ or cross-section$ or longitudinal$ or follow-up-stud$ or review$ or meta-analys$ or incidence$) |
